# The Differential Diagnosis of the Cause of Sudden Unconsciousness

**Published:** 1882-09-20

**Authors:** R. O. Beard


### THE DIFFERENTIAL DIAGNOSIS OF THE CAUSE OF SUDDEN UNCONSCIOUSNESS.

By R. O. BEARD, M. D.

(*Concluded from August Number.*)

URÆMIC POISONING

is a questionable cause of sudden unconsciousness, but certain cases are reported in which the usual prodromata are said to have passed unnoted, until the abrupt occurrence of convulsions and coma.

The convulsions are of the epileptiform type, and liable to remission. The presence of anasarca; the urinous odor of the breath; the dilated, indolent pupils; slow, stertorous respiration of a peculiarly labial character, distinguishable from the guttural sounds characteristic of cerebral hæmorrhage and compression; and the progressively falling temperature, reaching in some cases 91.5° F., furnish sufficient diagnostic evidence. If urinalysis is possible, the discovery of albumen and casts will settle the question of cause.

I cannot overlook the fact that a remarkable difference of opinion exists on the part of Professor Flint, as to the temperature of this condition. He claims that a marked elevation, sometimes ranging as high as 105° F., is observed in these cases. This statement is supported in Ziemssen's Cyclopœdia, where its explanation is attempted by a reference to the epileptiform nature of the convulsions, and the correspondingly high temperature of true epilepsy. But, although *epileptiform*, the convulsions are not *epileptic*, and the analogy fails in view of the wide difference in both causes and effects of the two diseases.

Neither can the excessive muscular exertion thus induced be regarded as sufficient cause for this reported elevation. Considering the fact that all other toxic agents which induce coma, among which uræmic poison may properly be classed, invariably occur with *lower* temperature, the above statement appears still more doubtful. In the apparent absence of post-mortem facts to sustain the clinical evidence, I would venture to suggest the occasional occurrence of meningitis as a complication of uræmia, and a possible cause of high temperature, and hence of these conflicting observations.

ASPHYXIA

is properly ranked among comatose conditions of toxic origin, but is too familiar to need anything beyond mention in this connection.

It is well-defined as "a suspension of animation, due to the non-conversion of venous into arterial blood." The indications point to primary disturbance in the lungs rather than the brain.

ALCOHOLIC COMA

would appear, at first thought, to be of all forms of unconsciousness the most easily diagnosticated, and yet no other condition has been so fruitful of mistake, and of injury as the result of error.

A medical verdict of "intoxication" has too often consigned an innocent sufferer from far more serious ills to a course of treatment which involved either irretrievable physical mischief, or a form of moral injury equally impossible of repair.

The odor of alcohol thrown off by the lungs is a valuable aid to diagnosis, but even in the presence of this indisputable evidence of inebriation, it should be remembered that accident incurred while in a drunken state may cause concussion or compression of the brain, or that cerebral hæmorrhage may supervene in this condition. The absence of paralysis, the presence of a complete muscular relaxation and anæsthesia, and the state of the pulse, the respiration and the skin, are all worthy of note, but the condition of the temperature and the pupils furnish the most valuable and distinctive evidence. A low temperature, sometimes markedly low, is not absolutely peculiar to acute alcoholism, as opposed to other forms of unconsciousness, but in none, save that of uræmic poisoning, is there so frequent and so considerable a fall.

Dr. Wm. MacEwen, of Glasgow, Scotland, records a thermic test in fifty cases, noted under many varying circumstances, which shows a downward range of temperature from 97.9° to 93.4° F. These figures represent rectal temperatures, which, according to Wunderlich, should be reckoned from a half to one degree higher than in the axilla. It may, therefore, be stated that in alcoholic coma the temperature has a latitude of 5° below normal. The same observer claims the demonstration of an absolutely pathognomonic sign in the condition of the pupils, which, as far as my observation and inquiry have extended, has proved a reliable aid in the diagnosis of doubtful cases and the exclusion of any coincident injury or disease.

In alcoholic coma *undisturbed*, he states that the pupils are *invariably contracted*, but that on the application of any external stimulus, sufficient to partially arouse the patient they *at once dilate*, again relapsing into a contracted state as unconsciousness redeepens. Dr. MacEwen reports the observation of fifty cases, of which number forty-eight answered perfectly to the test, contraction recurring in from five to thirty minutes after stimulation. The two exceptions to this rule proved, on subsequent inquiry, to have formerly suffered with disease of the iris, which had resulted in complete fixity of the pupils. Since the publication of these items I have endeavored, as opportunity offered, to test their accuracy, and in some eight cases I have noted the behavior of the pupils has been in perfect accord with the facts stated. This alternation is, I believe, characteristic of no other comatose condition, and is, therefore, if supported by further investigation, an invaluable diagnostic sign.

OPIUM NARCOSIS

is so rapidly fatal in its course that it especially demands prompt recognition. In the early stages of its action the patient can be momentarily aroused. The odor of opium on the breath can usually be detected. The complete muscular relaxation, the suppression of all secretions except that of the skin, which is very profuse, the usually persistent vomiting, the markedly contracted and insensible pupils and slightly reduced temperature, with other general symptoms, should be borne in mind.

The symptoms attending the narcosis of other soporific poisons are similar to the above. In the case of chloral, dilatation instead of contraction of the pupils is observed when poisoning results from the slow cumulative action of the drug.

In closing the discussion of these causes of sudden unconsciousness, let me refer briefly to those surgical conditions known as

CEREBRAL CONCUSSION, CONTUSION AND COMPRESSION.

Any attempt to differentiate their peculiar symptoms is necessarily limited by the fact that the occurrence of either of these distinctive forms of injury uncomplicated with one or both of the others is exceedingly rare. Indeed, Dr. Bryant explicitly stated that "concussion and contusion of the brain are *always* associated in fatal cases;" and the Reports of Guy's Hospital, London, for fifteen years, record "no case of death from concussion without change of brain structure."

Compression doubtless often happens alone. The possibility of an apoplectic or epileptic seizure occurring in a position likely to provoke a suspicion of accident, or even inducing a fall, should always be remembered."

*Cerebral Concussion*, pure and simple, is believed by many surgeons to be little more than a momentary disturbance, and certainly cases on record in which coma has persisted for hours or even days can hardly be regarded as uncomplicated. The loss of consciousness resulting is usually immediate and complete.

*Cerebral Contusion* is defined to be "a sudden and violent attrition of the brain substance, attended with more or less laceration and effusion of blood," generally in the form of minute hæmorrhagic points. It does not necessarily involve injury, actual or apparent, to the calvaria. Its symptoms, as apart from those of concussion and compression, are not well differentiated.

*Cerebral Compression*, of traumatic origin, may depend upon the pressure of fragments of the outer or inner tables of the skull, or of a blood-clot formed beneath the seat of injury.

A short interval usually elapses between the infliction of the injury and the appearance of the profound unconsciousness which follows. The general identity of its symptoms with those attendant upon cerebral hæmorrhage in its idiopathic form, indicates that the results are substantially the same in the two cases. For the general symptoms observable in these surgical cases, as well as in the other foregoing morbid conditions, I would refer to the summarized table appended to this sketch, which offers a readier means of rapid comparison than it would be possible to give in any other form.

It cannot but be observed that, in many cases, the means of differentiation we possess are at present very limited, and even those most clearly defined are often clouded by varying complications.

There only remains to allude briefly to the *modus operandi* of the causes which result in this general effect of unconsciousness, and on this important but obscure question I shall venture but a few simple suggestions.

The post-mortem evidence recorded throws but little light upon the working of these causes; but, applying the few facts thus obtained to the clinical history we possess, the indications seem to point strongly to *one* pathological condition necessary to the production of unconsciousness in any of its forms, viz.: a deficiency of arterial or oxygenated blood in the vessels of the brain. If this point be well taken, we have at once an explanation of the difficulty we meet in attempting to differentiate these conditions, in the fact that, though induced by exciting causes of widely varying nature, in many varying ways, and in greatly varying degree, the ultimate result, manifested in the loss of consciousness, is in each and all the same.

Thus, in syncope, probably the simplest form of unconsciousness, we find an anæmia of the cerebral cortex, whether caused by arrest of the general circulation, by profuse hæmorrhage from other parts, or by a chronic anæmic condition. In catalepsy and cerebral hysteria, the symptoms point to the same result, depending upon deranged nervous function, probably manifesting itself through the vaso-motor system by inducing a tonic contraction of the cerebral arterioles. In concussion of the brain, the theory of vascular paralysis affecting the vessels of the cortical portion of the cerebrum and producing arterial anæmia, with consequent venous stasis, offers the best explanation of the phenomena observed; and, seeing that we have no knowledge of the long continuance of such vascular paralysis, it accords well with the opinion that true concussion is but of short duration.

Insolation furnishes us with an instance of more complicated action, but it is probable, as Niemeyer observes, that the febrile condition of the blood, caused by the excessive heat and exposure, involves the loss of its oxygenating power.

Epilepsy has hitherto been very obscure in its pathology, but leading neurologists support the theory that the coma is due to general cerebral anæmia, while they refer the attendant convulsions to the excitation of a "convulsive center" by means of a local hyperæmia or congestion. This question doubtless requires further demonstration.

In the toxic conditions we have reviewed, we can distinctly trace the disturbance of cerebral function to the influence of the toxic agent, either directly upon the blood, as in uræmia and asphyxia; upon the heart and vessels through the agency of the nervous system, as in opium narcosis; or upon both, as in alcoholic intoxication.

By whatever mode of action, the results seem to be in each instance the same—that is, either the destruction or impairment of the oxygenating power of the blood, or an interference with the circulation in the cerebral substance.

In cerebral and meningeal hæmorrhage, and in traumatic compression, a similar end is reached by means of mere mechanical pressure.

Dr. Bryant observes that "unconsciousness in these cases, whether from injury or hæmorrhage, appears as soon as commencing intracerebral pressure impinges upon the capillaries of the cortex," and it is probable that it ensues even before that point is reached.

The rupture of an abscess into the brain substance has the same mechanical effect, and embolism, obstructing the cerebral arteries, induces partial pressure, and causes a more circumscribed anæmia, which, in its turn, accounts for the slighter and more transient symptoms.

The uncertainty which evidently surrounds this question in the minds of scientific observers, and leaves room for these imperfect suggestions, offers a wide field for patient investigation and research.—*Chicago Med. Jour. and Exam'r.*

